# Clinical Profile and Risk Factors of Group B Streptococcal Colonization in Mothers from the Eastern District of China

**DOI:** 10.1155/2022/5236430

**Published:** 2022-08-29

**Authors:** Jin Jiao, Weiwei Wu, Feng Shen, Zhongyuan Liu, Huiru Zhou, Gang Fan, Yuxia Zhou

**Affiliations:** ^1^Department of Clinical Laboratory, Maternal and Child Health Hospital of Shandong Province, Jinan, China; ^2^Shandong Pharmacists Association, Jinan, China; ^3^Department of Clinical Laboratory, Shandong Provincial Hospital Affiliated to Shandong First Medical University, Jinan, China

## Abstract

**Objective:**

The main aim of this study was to determine the prevalence, capsular genotyping, antimicrobial susceptibility, and associated factors of colonizing Group B *Streptococcus* (GBS) in pregnant women admitted to a hospital in Jinan, East China.

**Methods:**

Demographic data, clinical characteristics, and vaginal and rectal swabs were obtained from a group of expecting mothers subjected to GBS screening at the late stage of pregnancy who went into labor over the period from November 2019 to October 2020. Identification of GBS and determination of antimicrobial resistance patterns were performed using a BD Phoenix-100 system. Capsular genotypes were analyzed using polymerase chain reaction and the associated factors were evaluated via logistic regression.

**Result:**

A total of 2761 pregnant women were recruited for this study. The GBS colonization rate was 6.70% (185/2761). Among the 172 GBS strains examined, all were susceptible to vancomycin and linezolid. Resistance was the highest for erythromycin (80.2%), followed by clindamycin (75.0%), levofloxacin (65.1%), and tetracycline (57.6%). The most common serotype identified was Ia (61.0%), followed by III (29.7%), VI (4.6%), II (3.5%), VII (0.6%), and a nontypeable strain. Risk factors for maternal GBS colonization included maternal age (older than 30 years) (OR = 1.913 (1.662, 2.478)), gestational age at birth (average gestational age) (OR = 1.992 (1.445, 2.746)), and prelabor rupture of membrane (OR = 3.838 (1.619, 9.099)).

**Conclusion:**

The prevalence of GBS was relatively low. The maternal age was a factor associated with GBS colonization. Subjects showing GBS positivity during late pregnancy were prone to prolonged rupture of the membrane (PROM) and birth at lower a gestation age than the GBS-negative group. Penicillin could still be used as the first agent of choice for intrapartum antibiotic prophylaxis (IAP).

## 1. Introduction

Group B *Streptococcus* (GBS), also known as *Streptococcus agalactiae*, is a gram-positive bacterium with the ability to transfer infection from mother to fetus, causing neonatal sepsis and meningitis [1–3]. The latest worldwide systematic review disclosed rates of GBS colonization in pregnant women ranging from 2.0 to 32.0% [4]. Significant differences in the frequency of maternal colonization have been reported according to region, ethnicity, and socioeconomic characteristics [5].

GBS infection occurs through either vertical or ascendant pathways during delivery or after membrane rupture [5]. Ascending infection can cause maternal, fetal, and early-onset neonatal disease (days 0–6) leading to maternal death, stillbirth, and/or neonatal death [6–8]. Moreover, survivors of neonatal or infant GBS disease may suffer from neurodevelopmental impairment. Maternal colonization of GBS in the genitourinary tract is the primary risk factor for early-onset GBS disease (EOGBS) [1]. A recent meta-analysis reported that with the high absolute number of births and thus newborns exposed, Asia had the highest number of EOGBS (95 000) cases (UR, 53–143,000), of which China accounted for 25,000 (UR, 0–59,000) cases [9].

Intrapartum antibiotic prophylaxis (IAP) based either on microbiological screening or clinical risk factors is effective for the EOGBS disease but not late-onset GBS, stillbirth, and preterm birth [9]. Vaccine candidates under development for GBS include protein-based formulations and serotype-specific polysaccharide-protein conjugates [9]. Therefore, the elucidation of serotype distribution in maternal and infant disease is crucial for the prevention of GBS infections. Although earlier reports have identified a number of potential risk factors for GBS colonization and outcomes of pregnancy—such as maternal age, impaired glucose tolerance, anemia, gravidity, parity, preterm birth, birth weight, and prelabor rupture of membranes—results across studies are inconsistent and the effect sizes for identified risk factors vary considerably among different countries [1], knowledge of the prevalence and serotype distribution of GBS and risk factors for GBS colonization should help in the selection of appropriate preventive measures. To date, few studies have been conducted on GBS infection in the Shandong province of China. Here, we aimed to investigate the colonization, capsular genotypes, and antimicrobial susceptibility patterns of GBS isolated from pregnant women admitted to the Maternal and Child Health Hospital of Shandong province. Associations of clinical and social aspects with GBS carriage were additionally explored. Our collective findings may contribute to the guidance of effective disease prevention practices targeting GBS.

## 2. Materials and Methods

### 2.1. Ethics

A cross-sectional study was developed at the Maternal and Child Health Hospital of Shandong province (Jinan, China). This study was approved by the Research Ethics Committee of the hospital (No. 2021-114).

### 2.2. Specimen Collection

A cross-sectional study was developed at the Maternal and Child Health Hospital of Shandong province (Jinan, China). We enrolled pregnant women with GBS screening results obtained during late pregnancy and obstetrics information at the Maternal and Child Health Hospital of Shandong province from November 2019 to October 2020. Vaginal and anal swabs were routinely collected from expecting mothers in two tubes at the participating maternity center. Swabs were transferred to the bacteriology laboratory within 1 h and inoculated separately onto chromogenic agar plates without preenrichment (Guangzhou Rhfay Biological Medical Technology Corp, Guangzhou, China). Drug sensitivity tests were based on streptococcus identification and drug-sensitive plates using the BD Phoenix-100 system (Becton, Dickinson and Company, New Jersey, America). Serotyping of GBS isolates was conducted via conventional multiplex PCR. The primers were designed according to the reference sequences and synthesized by An Hui General Biosystems (Chuzhou, China) [10, 11].

### 2.3. Study Variables

The main outcome variable was GBS colonization, which was defined as cultures from lower vaginal or rectal samples testing positive in the absence of symptoms and signs of infection. Potential influencing factors were selected a priori on the basis of literature review, age (years), anemia (Hb < 110 g/L), parity (number of births), gravidity (number of times of pregnancy), gestational age (weeks), prolonged rupture of the membrane (PROM), delivery method (vaginal birth or cesarean section), preterm birth, birth weight, fetal sex, and gestation age for GBS screening.

### 2.4. Data Analysis

Data were analyzed using the Statistical Package for SPSS version 26.0. Bivariate analysis was performed using Fisher's exact test to evaluate the association between risk factors and colonization of GBS in pregnant women. Logistic regression was performed to compute odds ratios (OR) with 95% confidence interval (CI). *P* values <0.05 represent a statistical significance.

## 3. Results

### 3.1. Prevalence Rate of GBS

Among the samples obtained from 2761 pregnant women, 185 (6.70%) tested positive for GBS colonization, of which 45 (24.3%) were vaginal swabs and 73 (39.5%) were rectal swabs. A total of 67 (36.2%) among 210 participants tested positive on both rectal and vaginal swabs. The age of pregnant women in this study ranged from 18 to 45 years (mean age of 30.28 years with a standard deviation of ±3.82). In addition, the GBS colonization rate varied over time. Specifically, the colonization rate was the lowest in April (spring) and the highest in January (winter).

### 3.2. Risk Factors for GBS Colonization in Late Pregnancy

The study cohort was divided into GBS-positive and GBS-negative groups. The demographic and clinical characteristics of the two groups are listed in Table 1. Bivariate analysis revealed no associations of anemia (Hb < 110 g/L), parity, gravidity, gestational age, delivery method, preterm birth, birth weight, fetal sex and gestation age for GBS screening with GBS colonization in late pregnancy (*P* > 0.05).

The rate of GBS carriage in participants aged 30 years and older was significantly higher than that in younger mothers (OR = 1.913 (1.662, 2.478)) (Table 2). The rate of PROM was markedly higher in the maternal GBS colonization group relative to non-GBS colonization (OR = 3.838 (1.619, 9.099)), along with a below average gestational age at birth (OR = 1.992 (1.445, 2.746)). We observed no marked differences in fetal sex, fetal birth weight, premature delivery, maternal anemia, delivery method, history of gestation and pregnancy, and gestational age of GBS screening between the groups.

### 3.3. Antibiotic Susceptibility Profiles

Among the 185 GBS strains, 172 (93.0%) were examined for antibiotic susceptibility using the BD Phoenix-100 system. All strains were susceptible to vancomycin and linezolid. Resistance was the highest for erythromycin (80.2%), followed by clindamycin (75.0%), levofloxacin (65.1%), and tetracycline (57.6%). One strain was nonsusceptible (NS) to penicillin, meropenem, amoxicillin, cefotaxime, and cefepime and another strain, to cefotaxime and cefepime (Table 3). Multidrug resistance (MDR) was detected in 73.8% (127/172) cases.

### 3.4. Serotypes of GBS

Among the 185 GBS strains, serotypes of 172 (93.0%) were determined via multiplex PCR. The most common serotype was Ia (105/172, 61.0%), followed by III (51/172, 29.7%), VI (8/172, 4.6%), II (6/172, 3.5%), and VII (1/172, 0.6%). One strain was nontypeable (1/172, 0.6%).

## 4. Discussion

A total of 2761 pregnant women with ages ranging from 18 to 45 years from Jinan (Shandong, China) were recruited for the study. The prevalence of GBS during late pregnancy was 6.7%, with Ia and III identified as the dominant serotypes. The maternal age was associated with GBS colonization. GBS positivity in women at the late pregnancy stage was more highly associated with the tendency of PROM and birth at low gestational age relative to the GBS-negative group.

Until recently, there has been uncertainty regarding the burden of GBS disease in Asia and limited epidemiological studies on maternal GBS colonization in mainland China are documented in the literature [9, 12]. The GBS colonization rate in late pregnancy of 6.70% in the current study was lower than that in Southern Asia (13%) and Eastern Asia (11%), distinct from other regional studies in China. For example, the prevalence of GBS colonization in Jinan was higher than 4.9% reported in Shenzhen, 3.7% in Shanghai, 4.16% in Nanjing, and 5.02% in Chengdu but lower than 21.8% in Hong Kong, 13.89% in Xiamen, 10.61% in Qingdao, 8.2% in Dongguan, and similar to 7.1% in Beijing, 6.4% in Jiangsu, and 6.1% in Liuzhou [12–18]. These inconsistencies may be attributable to a number of factors. GBS screening was not conducted for all pregnant women at the hospital, and we adopted the culture of chromogenic agar plates without preenrichment but not PCR, which could potentially lead to a lower estimated prevalence of GBS than the actual value.

GBS serotypes were determined with the aid of multiplex PCR in our study. Overall, five serotypes (Ia, II, III, VI, and VII) were identified, with prevalence rates ranging from 0.6% to 61.0%. Among these, 105 strains (61.0%) were genotyped as Ia, 6 (3.5%) as II, 51 (29.7%) as III, 8 (4.6%) as VI, and 1 (0.6%) as VII. Two isolates (0.6%) were not successfully genotyped using this methodology. The serotype distribution of GBS isolates identified in our cohort is similar to that described globally, with serotypes III, II, and Ia being the most common [19]. Our findings are distinct from a Chinese multicenter cohort study and a Korean research study in 2017–2019 [20], which showed that the predominant serotypes were type III (35.9%), Ia (22.5%), V (21.2%), and Ib (10.4%), and the predominant serotypes were V (22.7%), VIII (20.0%), and III (20.0%) [21]. One reason may be alterations in the prevalent serotype during different periods. In addition to different clonal expansions, horizontal transfer of capsular genes or capsular switching among different clones may represent other contributory factors [22–25]. Knowledge of serotype prevalence could be effectively used to inform vaccine design and facilitate subsequent monitoring of serotype replacement. According to the data from our study, a pentavalent vaccine (Ia, Ib, II, III, and V) and trivalent CPS vaccine (Ia, Ib, and III) would be able to cover 94.2% and 90.7% of GBS infections in pregnant women in Jinan, China [9].

For IAP and treatment of GBS infections, penicillin is the firstline antibiotic of choice [26]. Clindamycin, erythromycin, and levofloxacin are important alternatives for individuals allergic to penicillin [27]. Earlier reports reported the prevalence of resistance to erythromycin and clindamycin of 61.5% and 51.9% in Shanghai (2015), 77.5% and 68.3% in Jiangsu (2017–2019), and 76.23% and 58.21% in Vietnam (2016–2020) [14, 21, 28]. In our study, all GBS strains were susceptible to vancomycin and linezolid. However, the strains showed highest resistance to erythromycin (80.2%), followed by clindamycin (75.0%), levofloxacin (65.1%), and tetracycline (57.6%). Moreover, one strain was not susceptible to penicillin, meropenem, amoxicillin, cefotaxime, and cefepime and another, to cefotaxime and cefepime. Although our data on susceptibility to penicillin were inconsistence with previous findings on GBS colonization in pregnant women in China [14, 21], resistance rates to erythromycin and clindamycin were higher, supporting the proposal that individuals allergic to penicillin should be tested for antibiotic susceptibility of GBS isolates.

We additionally determined the essential risk factors associated with maternal GBS colonization. Bivariate analysis revealed no association of anemia (Hb < 110 g/L), parity, gravidity, gestational age, delivery method, preterm birth, birth weight, fetal sex, and gestation age for GBS screening with GBS colonization in late pregnancy (*P* > 0.05) while maternal age, gestational week of birth, and PROM were correlated with GBS colonization (*P* < 0.05). The lack of association of parity, gravidity, delivery method, birth weight, and fetal sex with maternal late GBS colonization was consistent with previous findings; however, data were inconsistent regarding maternal age, anemia, preterm birth, diabetes, and PROM [3, 8, 13, 18, 28, 29]. These discrepancies may be attributable to differences in demographics, sexual activity during pregnancy, cutoff points, and fitted model structures [1, 8].

Further, logistic regression was conducted to validate the relationships between maternal age, gestational age of birth, PROM, and GBS colonization. Consistent with some previous reports, older age was associated with greater susceptibility to GBS carriage [OR = 1.913(1.662, 2.478)] [11, 30]. In contrast, increasing age was significantly linked to lower rates of colonization in other research [31, 32]. We observed a greater likelihood of lower gestational age at birth for GBS-colonized mothers (OR = 1.992 (1.445, 2.746)). Moreover, consistent with data obtained from Inner Mongolia, but not Xiamen and Jakarta, GBS-colonized mothers were more likely to undergo PROM (OR = 3.838 (1.619, 9.099)) [11, 18, 30].

Our study has a number of limitations that should be taken into consideration. First, GBS screening was not conducted on all pregnant women admitted to the hospital. Second, we adopted the method of culture on chromogenic agar plates without preenrichment but did not perform PCR, and therefore, the prevalence of GBS in pregnant women was possibly lower than the actual value. To determine the risk factors of maternal GBS colonization, we only recruited those mothers who were subjected to GBS screening at >34 weeks gestation and gave birth at the Maternal and Child Health Hospital of Shandong province. Accordingly, the lack of sufficient information on established risk factors currently limits the comprehensive analysis of the efficacy of simulated risk-based strategies, highlighting the necessity of further investigation in this field.

The prevalence of GBS in pregnant women was low and within the range of earlier studies. Resistance of GBS isolates to erythromycin and clindamycin was high. Serotype distribution data should be helpful for guidance of the development of vaccines to prevent GBS disease and, more importantly, reduce stillbirths. The associated risk factors for GBS colonization and GBS burden of infants require further evaluation.

## Figures and Tables

**Table 1:** Demographic data and associations between risk factors and GBS colonization in pregnant women with gestation of >34 weeks.

Variables	*n*	*Maternal GBS colonization*		*P* value	OR (95% CI)
		Positive, *n* (%)	Negative, *n* (%)		
*Maternal age (years)*					
18–29	1300	81 (6.2)	1219 (93.8)	Ref	Ref
30–45	1461	104 (7.1)	1357 (92.9)	0.001	1.009 (0.990, 1.030)

*Hb*					
<110 g/L	781	45 (5.8)	736 (94.2)	Ref	Ref
≥110 g/L	1980	140 (7.1)	1840 (92.9)	0.099	1.014 (0.993, 1.036)

*Prelabor rupture of membrane*					
No	2291	146 (6.4)	2145 (93.6)	Ref	Ref
Yes	470	39 (8.3)	431 (91.7)	0.018	1.021 (0.992, 1.051)

*Premature delivery*					
No	2727	181 (6.6)	2546 (93.4)	Ref	Ref
Yes	34	4 (11.8)	30 (88.2)	0.235	1.058 (0.936, 1.197)

*Gestational age at birth*					
Below average gestational age	1205	110 (9.1)	1095 (90.9)	Ref	Ref
Above average gestational age	1556	75 (4.8)	1481 (95.2)	0.001	0.955 (0.935, 0.975)

*Delivery method*					
Vaginal	1986	125 (6.3)	1861 (93.7)	Ref	Ref
Cesarean section	775	60 (7.7)	715 (92.3)	0.072	1.016 (0.992, 1.040)

*Birth weight*					
Below average birth weight	1380	89 (6.4)	1291 (93.6)	Ref	Ref
Above average birth weight	1381	96 (7)	1285 (93)	0.598	1.005 (0.985, 1.026)

*Fetal sex*					
Boy	1413	94 (6.7)	1319 (93.3)	Ref	Ref
Girl	1363	95 (7.0)	1268 (93.0)	0.075	1.000 (0.981, 1.021)

*Gravidity*					
1	1308	88 (6.7)	1220 (93.3)	Ref	Ref
2	851	59 (6.9)	792 (93.1)	0.853	1.002 (0.979, 1.026)
3	380	26 (6.8)	354 (93.2)	0.938	1.001 (0.971, 1.033)
4	151	7 (4.6)	144 (95.4)	0.324	1.008 (0.942, 1.016)
5	53	4 (7.5)	49 (92.5)	0.816	1.009 (0.933, 1.091)
6	14	0 (0)	14 (100)	0.315	1.033 (0.919, 1.146)
7	4	1 (25)	3 (75)	0.147	1.244 (0.706, 2.190)

*Parity*					
Primiparous	1829	127 (6.9)	1702 (93.1)	Ref	Ref
2^nd^ parity	889	56 (6.3)	833 (93.7)	0.529	0.993 (0.972, 1.014)
3^rd^ parity	42	2 (4.8)	40 (95.2)	0.581	0.977 (0.912, 1.047)
4^th^ parity	1	0 (0)	1 (100)	0.785	0.931 (0.919, 0.942)

*Gestational age for GBS screening*					
<35 weeks	79	7 (8.9)	72 (91.1)	Ref	Ref
35–36 weeks	825	56 (6.9)	759 (93.1)	0.509	0.979 (0.911, 1.051)
36–37 weeks	1171	71 (6)	1104 (94)	0.315	0.970 (0.904, 1.041)
37–38 weeks	451	33 (7.3)	421 (92.7)	0.620	0.983 (0.913, 1.058)
>38 weeks	235	18 (7.6)	220 (92.4)	0.711	0.986 (0.912, 1.066)

**Table 2:** Risk factors for GBS colonization in pregnant women: binary logistic regression analysis.

Factors		*B*	SE	*P*	OR (95% CI)
*Maternal age (years)*	18–29	0			
	30–45	0.068	0.158	0.006	1.913 (1.662, 2.478)

*Prelabor rupture of the membrane*	No	0			
	Yes	0.158	0.440	0.002	3.838 (1.619, 9.099)

*Gestational age at birth*	Above average gestational age	0			
	Below average gestational age	0.689	0.164	0.001	1.992 (1.445, 2.746)

*B*: regression coefficient; SE: standard error; *P*: *P* value; OR: odds ratio. Average gestational age is 39 weeks 4 days.

**Table 3:** Antibiotic susceptibility profiles of 172 GBS isolates from pregnant women.

Antibiotic	Total	*S* (%)	*I* (%)	*R* (%)	NS (%)
Amoxicillin	172	171(99.4)	NA	NA	1(0.6)
Erythromycin	172	34(19.8)	0(0.0)	138(80.2)	NA
Clindamycin	172	40(23.3)	3(1.7)	129(75.0)	NA
Tetracycline	172	73(42.4)	0(0.0)	99(57.6)	NA
Chloramphenicol	172	149(86.6)	0(0.0)	23(13.4)	NA
Levofloxacin	172	59(34.3)	1(0.6)	112(65.1)	NA
Penicillin	172	171(99.4)	NA	NA	1(0.6)
Cefotaxime	172	170(98.8)	NA	NA	2(1.2)
Cefepime	172	170(98.8)	NA	NA	2(1.2)
Meropenem	172	171(99.4)	NA	NA	1(0.6)
Vancomycin	172	172(100.0)	NA	NA	0(0.0)
Linezolid	172	172(100.0)	NA	NA	0(0.0)

*S*: susceptible, *I*: intermediate, *R*: resistance, NS: nonsusceptible, NA: not applicable.

## Data Availability

The datasets used and analyzed during the current study are available from the corresponding author on reasonable request.
